# Surgery of the Intestines

**Published:** 1894-06-16

**Authors:** 


### PROGRESS IN SURGERY.

### SURGERY OF THE INTESTINES—(*continued*).

**Embolism of Mesenteric Artery.**—An uncommon cause of symptoms of obstruction is exemplified by a case published[13] by Dr. Munro showing the results of embolism of branches of the inferior mesenteric artery. The symptoms produced by embolism in the mesenteric vessels are similar in character, whether the branches of the superior or of the inferior artery are affected. In both intestinal obstruction is produced in the section of bowel which is more or less completely shut off from its blood supply, and the condition is much the same as that found in paralysis of the intestine, the result of injury received in a hernial protrusion. A similar case under the care of Mr. McCarthy has been published,[14] in which acute intestinal obstruction resulted from embolism of the superior mesenteric artery, and in which when abdominal section was performed six days after the commencement of the illness, perforation of the bowel with escape of contents into the peritoneal cavity had already taken place. Dr. Munro on opening the abdomen found the following conditions: There was a mass in the left iliac fossa due to a large decolorised infarction in the mesentery of the sigmoid flexure. There were two infarcts also in the mesentery of the small bowel; these were smaller, and retained for the greater part the dark colour of the blood and thus were more recent than that in the mesentery of the sigmoid flexure. The infarct in the sigmoid flexure was adherent to the abdominal wall and to the iliac fossa. The ascending, descending, and transverse cola were enormously distended and very dark in colour; the walls appeared to be thinned by the distension to which they had been subjected, and they had a doughy feel to the touch. The caput coli was also enormously distended, but otherwise not so much altered. The small intestines were red and deeply injected, which, together with the more recent appearances of the infarcts, proves that the inferior mesenteric artery was first affected. The patient progressed favourably for twenty-four hours, but then rapidly sank and died. Dr. Munro has taken the following from articles by Gerhardt and Kussmaul giving the diagnostic points of these cases: (1) A source of origin for the embolus; (2) profuse, even exhausting hæmorrhage from the bowels; (3) severe colic-like pains in the abdomen; (4) tension and tympanitic swelling, sometimes very marked; (5) considerable and rapid reduction of temperature; (6) demonstration of embolism in some of the other arteries; and (7) demonstration by palpation of infarcts between the mesenteries. In the case referred to nearly all these classical symptoms were present, and number 7 in so marked a degree that its firmness and immobility simulated a tumour and so led to operation.

**Excision of the Cæcum for Malignant Growth.**—Three successful cases are reported: In Dr. Ilott's[15] case the ileum and colon were approximated laterally by means of Senn's plates, but in the cases reported by Milton[16] and Sendler[17], an end-to-end anastomosis was effected after the colon had been sufficiently closed to reduce it to the size of the ileum.

An unusual case is published[18] by Mr. James Berry, in which the sigmoid flexure had become enormously distended, ulcerated and perforated as the result of simple chronic constipation. There was no stricture or other mechanical cause of obstruction. The cæcum, and ascending, transverse and descending cola were not distended or hypertrophied. The sigmoid flexure formed a huge sac, eighteen inches in circumference at its widest part. It occupied most of the left side of the abdomen and reached upwards as high as the spleen and liver, to both of which it was attached by old adhesions. The patient was a man seventy-three years of age, and was admitted to hospital in a state of collapse. For many years he had suffered from habitual constipation, which had been absolute for the previous nine days. Vomiting had begun on the day before admission. Symptoms of perforation had developed a few hours before admission, and death ensued soon after. Mr. Berry regarded the dilatation as purely passive, like the similar dilatation of the stomach.

12 Brit. Med. Journ., Jan. 13, 1894. 13 Lancet, Jan. 20, 1894. 14 Lancet, Mar. 22, 1890 (Mirror of Hospital Practice). 15 Lancet, Mar. 3, 1894, p. 538. 16 Lancet, Feb. 24, 1894, p. 468. 17 Brit. Med. Journ., Jan. 27, 1894; and Münch. Med. Woch, Jan. 2, 1894. 18 Lancet, Feb. 10, 1894, p. 335.

